# Incidence and Intensity of Pain Following Endodontic Treatment by Different Instrumentation Techniques in Teeth With Periapical Lesion

**DOI:** 10.7759/cureus.35354

**Published:** 2023-02-23

**Authors:** Payal Kundgulwar, Dr. Abhinay Agarwal, Madhura Pawar, Krishna Biswas, Chirag Joshi, Ashok Galav, Ramanpal Singh

**Affiliations:** 1 Conservative Dentistry and Endodontics, Teerthanker Mahaveer Dental College and Research Centre, Moradabad, IND; 2 Conservative Dentistry and Endodontics, Teerthanker Mahaveer Dental College and Research Center, Moradabad, IND; 3 Pedodontics and Preventive Dentistry, Dr. Dnyandeo Yashwantrao (D.Y. Patil Dental College & Hospital), Pune, IND; 4 Dentistry, All India Institute of Medical Sciences, Guwahati, IND; 5 Oral and Maxillofacial Pathology, Rungta College of Dental Sciences and Research, Bhilai, IND; 6 Oral Medicine and Oral Radiology, Dental College Azamgarh, Azamgarh, IND; 7 Oral Medicine and Radiology, New Horizon Dental College and Research Institute, Bilaspur, IND

**Keywords:** rotary, endodontics, postoperative pain, root canal treatment, visual analogue scale

## Abstract

Aim and objectives: This study was carried out to compare the effectiveness of hand and rotary instrumentation techniques on postoperative pain in asymptomatic necrotic premolars with periapical lesions and instrumented by a modified step-back technique using a K file, crown down by continuous rotary motion technique using ProTaper Universal (Dentsply Mailefer, Ballaigues, Switzerland), and with the reciprocation technique using WaveOne (Dentsply Sirona, Charlotte, NC, USA).

Materials and Methods: For this study, 66 premolars with single roots and canals were chosen. The procedure was completed in a single visit. Following access opening, the working length was initially determined using an apex locator and then confirmed after inserting K file #10 by radiograph. The canal was cleaned and shaped using a grouping system. After the master apical preparation, the canal was dried by paper point and obturated with gutta-percha and AH plus sealer, an epoxide-amine resin pulp canal sealer. In order to confirm the obturation, a radiograph was taken. After that, a permanent restoration material was used to seal the access cavity. Following that, patients to whom the visual analog scale (VAS) had already been explained were contacted by phone at six, 12, 24, and 48 hours.

Results: In this study, compared to a stainless steel instrumentation technique, WaveOne instrumentation caused more noticeable pain. The results of the current study showed that, on average, postoperative pain scores decreased over the course of the 12 to 48-hour period, reaching a minimum or a maximum at 48 hours (p<0.01).

Conclusion: Postoperative pain was produced by all instrumentation methods used in the study. In comparison to ProTaper and WaveOne, instrumentation using the modified step-back technique with K files caused less pain, especially over the course of a 24-hour period.

## Introduction

One of the most completely avoidable side effects of root canal therapy is pain. The extent of tissue damage and pain intensity appear to be related. Inter-appointment or postoperative pain is brought on by periradicular inflammation caused by debris extrusion and is mediated by the nociceptors' G protein-coupled receptors being stimulated by the substances calcitonin, gene-related peptides, and substance P [1,2]. Allodynia, hyperalgesia, and spontaneous pain are the results of this. Additionally, central sensitization starts when there is a flood of C-fibre inputs that is strong enough and lasts long enough. The cleaning and shaping of the root canal, which eliminates inflammatory and necrotic tissue are one of the most important steps in endodontic treatment. In the majority of cases, extrusion frequently contains necrotic tissue, dentine fragments, microorganisms, pulp tissue, and irrigants.

Diseases of the dental pulp and periradicular area are treated using root canal therapy. Single vs. multiple-visit endodontic management has been a topic of discussion ever since single-visit endodontic treatment first became available [3]. Supporters of single-visit endodontic treatment emphasize the time and money savings. Additionally, any mishaps that might occur in the middle of the visits-such as leakage through a temporary restoration in between appointments missed appointments, and reinfection-are prevented [4]. Even though the instrumentation is within the root canal's margins, debris extrusion is inevitable [1]. Instead of flaring the root canals using pull-and-push motions, the modified step-back preparation uses hand K files with reaming motion in the coronal third and circumferential filing in apical areas. These motions help to suspend debris in irrigation solutions and tend to lessen debris extrusion [5]. The ProTaper (Dentsply Mailefer, Ballaigues, Switzerland) has no radial land, a convex triangular cross-section, and a variable taper along its length. These instruments' cross-sections don't provide enough room for debris to build up, and their variable taper, which functions like a piston to force debris out the apex, prevents accumulation. When rotating at the release angle, the flutes in the reciprocating single-file technique tend to push debris into the apical area rather than remove it. Additionally, the WaveOne (Dentsply Sirona, Charlotte, NC, USA) method uses a large, moderately rigid single file with a greater taper that immediately reaches the apex. In some circumstances, reciprocal tools with force concentration at the apex are used to reach the apical working length. This causes an active piston to push debris out of a patent apical foramen [6].

Thus, the present in vivo study was undertaken to evaluate the clinical assessment of incidence and intensity of postoperative pain in asymptomatic necrotic premolars with periapical lesions following single-visit root canal treatment by hand instruments, continuous rotary, and reciprocation instrumentation.

## Materials and methods

Before beginning treatment, each patient's written informed consent was obtained, and ethical clearance was obtained from the Institutional Review Board of Teerthanker Mahaveer Dental College and Research Centre (approval no. TMDCRC/IEC/19-20/CDE5).

Patients aged 25 to 40 years with an asymptomatic necrotic single-rooted premolar having a periapical lesion less than 2 cm in size without a sclerotic border on Intraoral periapical radiographs (IOPAR) were included in the study. Patients taking corticosteroids, opioids, or non-steroidal anti-inflammatory drugs (NSAIDs); teeth with calcified canals; an immature apex; and patients undergoing re-treatment, were excluded. Patients with pre-operative pain, root canal curvature greater than 30°, or percussion tenderness were also excluded from the study.

Sixty-six individuals with single canals and single-rooted maxillary and mandibular premolars were randomly selected and divided into three groups of 22 teeth in each group. Under rubber dam isolation, the entire root canal therapy procedure was completed in a single visit. The working length was determined using the apex locator Root ZX mini (J. Morita Corp., Osaka, Japan) after the access was opened, and it was then confirmed by radiograph after inserting K file #10. Based on grouping, the canal was cleaned and shaped. Along with the usual irrigation procedure, irrigation with 3% sodium hypochlorite (NaOCl) and normal saline was also carried out. Finally, the canal was rinsed for one to two minutes with ethylenediamine tetraacetic acid (EDTA).

The following instrumentation technique was used to clean and shape the root canal: K file's modified step-back method for Group 1, a crown-down technique utilizing ProTaper Universal in a continuous rotary motion for Group 2, WaveOne reciprocation technique (#20 to #45) for Group 3.

Following preparation, the canal was dried with a paper point and obturated with gutta-percha and AH plus sealant (epoxide-amine resin pulp canal sealer) using a cold lateral compaction technique. To confirm the obturation, a radiograph was taken. After that, a durable restoration material was used to seal the access cavity. There was no occlusal reduction done. A second clinician then called patients who had already been told about the visual analog scale (VAS) at six, 12, 24, and 48 hours. Patients were notified and recalled if full coverage restoration was necessary after 10 days. The evaluation standards were noted. The patients were instructed to take ketorolac 10 mg (SOS) if necessary and to inform the doctor if the pain became intolerable or unavoidable. Statistical analysis was done after data collection.

Pain evaluation criteria involved the presence or absence of pain recorded as a score of 1 to 4 based on the Heft-Parker VAS. The patient was asked to make a mark on a line indicating how much pain they were experiencing (Table 1).

**Table 1:** Pain evaluation criteria

Score	Score 1 (0-6)	Score 2 (7-54)	Score 3 (55-114)	Score 4 (114 above)
Pain intensity	No pain	Mild pain	Moderate pain	Intense pain

Data were statistically analyzed using the Statistical Package for Social Sciences (SPSS) version 26.0 (IBM Corp., Armonk, NY, USA). By using the chi-square test, the frequencies of categories were compared among groupings. A p-value <0.05 was considered to be statistically significant for all the statistical tests.

## Results

Intergroup comparison

**Figure 1:**
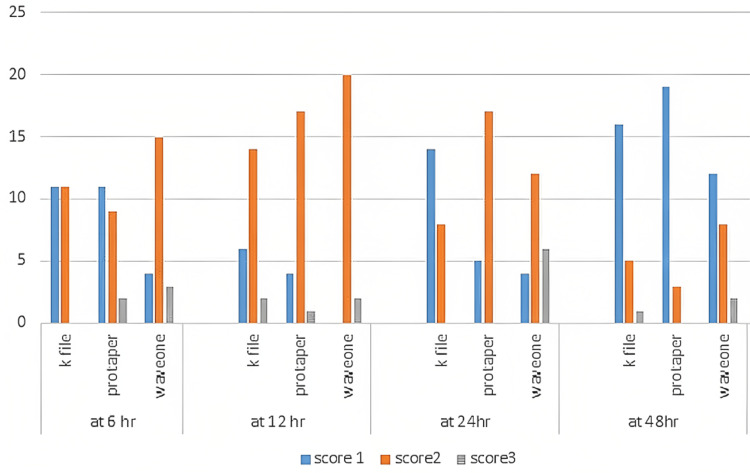
Intergroup comparison

There was a statistically non-significant difference at the sixth, 12th, and 48th hour (p>0.05). Whereas a statistically highly significant difference (p<0.01) with higher frequency for score 2 in Groups 2 (ProTaper) & 3 (WaveOne) with score 1 in Group 1 (hand K file) was found at the 24th hour (Figure 1).

Intragroup comparison

There was a statistically significant difference seen for the incidences of pain between the time intervals (p<0.05) with a higher incidence of score 1 at the 48th hour, and score 2 at the 12th hour when instrumentation was carried out using a K file. In the ProTaper group, a statistically highly significant difference was seen (p<0.01) with higher incidence for score 1 at 48 hours, score 2 at 12 hours, and 24 hours. In the WaveOne group, there was a statistically highly significant difference seen (p<0.01) with higher incidence for score 1 at 48 hours, and score 2 at 12 hours (Tables 2, 3, 4).

**Table 2:** Pain scoring at different time intervals with K File

Group	K file				
Pain Score	1	2	3	Chi-square value	p-value
6 hours	11	11	0	8.169	0.086
12 hours	6	14	2	7.059	0.133
24 hours	14	8	0	23.210	0.001
48 hours	16	5	1	5.949	0.203

**Table 3:** Pain scoring at different time intervals with ProTaper

Group	ProTaper Universal				
Pain Score	1	2	3	Chi-square value	p-value
6 hours	11	9	2	8.169	0.086
12 hours	4	17	1	7.059	0.133
24 hours	5	17	0	23.210	0.001
48 hours	19	3	0	5.949	0.203

**Table 4:** Pain scoring at different time intervals with WaveOne

Group	WaveOne				
Pain Score	1	2	3	Chi-square value	p-value
6 hours	4	15	3	8.169	0.086
12 hours	0	20	2	7.059	0.133
24 hours	4	12	6	23.210	0.001
48 hours	12	8	2	5.949	0.203

## Discussion

A single visit or multiple visits may be necessary for endodontic treatment [7]. Studies on postoperative pain and healing rates show that whether treatment is completed in one visit or multiple visits, the outcome is the same. Necrotic premolars with periapical lesions were included in the current study because, according to a systemic review, the healing rate for an infected tooth is the same whether it is treated in a single visit or over the course of multiple visits [8].

Even though analgesics are frequently provided, pain following endodontic treatment still poses a serious problem for patients [9]. The incidence of post-endodontic pain, which ranges from 82.9% to 10.6% in the reports, is highly variable [10,11]. These variations in the outcome may be the result of differences in the use, taper, cross-section, alloy type, cutting-edge design, flexibility, kinematics, tip type, and the number of files used concepts, which vary with file systems and operators' abilities. As the incidence and intensity of pain significantly decreased within the first two days, a VAS was used to record the intensity of pain at a maximum 48-hour time interval in this study [12].

In this study, it was discovered that K-file instrumentation had a lower prevalence of postoperative pain 24 hours after root canal therapy than ProTaper and WaveOne (p<0.01), but at six hours, 12 hours, or 48 hours, there was no statistically significant difference between the groups. In the current study, circumferential filing in the apical regions and modified step-back preparation in the coronal third was used in place of pulling and pushing motions to flare the root canals. Instead of causing debris to be extruded from the canal, these movements help to suspend debris in irrigation solutions, which lowers the likelihood of postoperative pain [5]. According to a study by Ahmed et al., patients treated with the ProTaper rotary and those treated with the manual step-back technique did not experience any appreciable differences in the frequency of pain [13]. Similar findings were reported in a randomized clinical trial conducted by Kashefinejad et al., which claimed that there was no difference between the reciprocal and rotational instrumentation approaches in terms of the frequency or severity of postoperative discomfort [14]. In contrast, the rotational instrumentation showed slightly higher pain scores than the reciprocation method. In a randomized control trial, Shokraneh et al. found that patients who underwent instrumentation with the WaveOne file as opposed to the ProTaper Universal and hand files experienced significantly less pain [15].

At 48 hours, the incidence and severity of pain were lower in the group instrumented using K files, while mild pain was noted at 12 hours (p<0.05). According to Pasqualini et al., manual K files for glide path files resulted in less postoperative pain than mechanical preparation with path files [16]. WaveOne instrumentation in the current study caused a more significant pain and inflammatory response in relation to K1. A smaller releasing angle and a wider cutting angle are the potential mechanisms that lead to more severe postoperative pain after using the WaveOne instrument. Additionally, the amount of debris removed is less with a reciprocating file than with a continuous rotation due to debris entrapment within the flutes, increasing frictional stress and torque demand [17]. The results of the current study showed that mean postoperative pain scores decreased from 12 to 48 hours after surgery, with no or very little pain at 48 hours (p<0.01). Patients' varying emotional states and levels of pain may have an impact on how well they respond to treatment.

Age, sex, the condition of the pulp and periradicular tissue, preoperative pain, and instrumentation technique are some of the variables that may affect how postoperative pain manifests [18]. The operator has control over elements like irrigation, obturation, and canal instrumentation. By creating a clean, well-shaped canal and limiting the extrusion of canal contents during treatment, it is thus possible to prevent postoperative pain.

The study's limitations include the requirement that future research concentrate on the method in use at the point of time as advancements in endodontic materials and methods may change the factors that influence treatment outcomes, such as postoperative endodontic pain.

## Conclusions

With a variety of techniques and materials, root canal treatment can be accomplished in a single visit as an alternative to multiple visits. This study showed that no patient required additional treatment and that all instrumentation techniques resulted in minimal pain and discomfort. In comparison to ProTaper and WaveOne, instrumentation using the modified step-back technique with K files caused less pain, particularly over a period of 24 hours. The study also found that when pain does occur, it is anticipated that it will lessen in intensity after 12 hours of root canal therapy. Dental procedures must include pain management from the very beginning to avoid exacerbation of the pain. Patients receiving dental treatment should be warned about the possibility of pain following root canal therapy and advised to take analgesics. Different instrumentation methods can be used in new studies to assess their impact on postoperative endodontic pain.
